# Identification of symptomatic carotid plaque by CTA-based radiomics: a multicenter study

**DOI:** 10.3389/fneur.2026.1750076

**Published:** 2026-01-21

**Authors:** Wei Zhang, Zongmeng Wang, Qiaomei Xu, Xi Wang, Le Zhang, Rong Chen, Mingsha Liao, Jianquan Zhong

**Affiliations:** 1 Department of Radiology, Affiliated Hospital of North Sichuan Medical College, Nanchong, Sichuan, China; 2 Department of Radiology, Zigong First People’s Hospital, Zigong, Sichuan, China; 3 Medical Imaging Center, Second People's Hospital of Yibin, Yibin, Sichuan, China; 4 Department of Radiology, The Affiliated Hospital of Southwest Medical University, Luzhou, Sichuan, China

**Keywords:** carotid atherosclerosis, computed tomography angiography, ischemic stroke, radiomics, symptomatic plaque

## Abstract

**Objectives:**

To develop and validate a combined model integrating traditional clinical characteristics, imaging features and radiomic features based on head and neck computed tomography angiography (CTA) to predict ischemic events in ipsilateral cerebral vessels.

**Methods:**

In this multicenter retrospective study, 223 patients from 3 independent centers were divided into training set (*n* = 134), internal test set (*n* = 34) and external validation set (*n* = 55). Based on recent symptoms (presence or absence of ipsilateral cerebral ischemia), patients were categorized into symptomatic group (*n* = 110) and asymptomatic group (*n* = 113). The traditional clinical characteristics, imaging features and radiomic features of all patients were collected. The traditional quantitative variables independently related to symptomatic carotid plaque were identified using univariate analysis and multivariate logistic regression analysis, and the intraclass correlation coefficient (ICC) and Least Absolute Shrinkage and Selection Operator (LASSO) regression analysis were applied to select robust radiomic features. Subsequently, three predictive models – the traditional model, radiomic model, and combined model integrating clinical, imaging and radiomic features – were constructed. Model performance was evaluated using receiver operating characteristic curves (ROCs) analysis, area under the curves (AUCs), calibration curves and decision curves analysis, and the accuracies of the models were verified in internal test set and external validation set.

**Results:**

Univariate analysis and multivariate logistic regression analysis showed that platelet distribution width (PDW) (odds ratio [OR] = 0.88; 95% confidence interval [CI], 0.80–0.97) and plaque ulceration (OR = 5.67; 95% CI, 2.86–11.23) were independently related to symptomatic plaque. Twelve radiomic features significantly related to symptomatic plaque were selected. The combined model demonstrated superior performance compared with both the radiomic model and the traditional model, the AUCs of the training set and internal test set were 0.819(95% CI: 0.749–0.888) and 0.785(95% CI: 0.620–0.950), and also demonstrated robust performance in external validation set (AUC: 0.868; 95% CI: 0.765–0.970).

**Conclusion:**

The Combined model demonstrated the highest diagnostic performance in identifying symptomatic plaque, which helps clinicians to analyze patients’ condition more comprehensively and provides additional value for identifying high-risk individuals and improving prognosis.

## 1 Introduction

Cerebrovascular disease (CVD) is a common neurological disorder that can be classified into ischemic and hemorrhagic types. Ischemic cerebrovascular disease (ICVD) is a leading cause of death and disability worldwide, characterized by a high recurrence rate, with the mortality rate of the first episode reaching approximately 30%. In addition, even among survivors, about 70% experience functional impairments such as hemiplegia and aphasia (1–3). Ischemic stroke (IS) is the most common type of cerebrovascular disease, accounting for more than 80% (3), with an annual recurrence rate is ranging from 9.6 to 17.7% (4), which has caused serious harm to patients’ lives and health, but also imposes a substantial economic burden on families and society. Carotid atherosclerosis is major risk factor for ICVD, and 20–30% of clinical cerebral ischemic events are related to carotid atherosclerotic stenosis (5). Therefore, carotid atherosclerotic plaque serves as a reliable indicator for predicting ischemic cerebral events. Symptomatic plaques refer to those plaques that cause clinical manifestations such as transient ischemic attack or ischemic stroke (6, 7). Early and accurate identification of these plaques can enable clinicians to implement timely interventions for high-risk patients better, reduce the incidence of cerebral ischemic events.

Head and Neck Computed Tomography Angiography (CTA) has the advantages of high spatial resolution, fast scanning speed, wide coverage, short imaging time and low radiation dose. This method can not only show the location, extent and degree of carotid artery stenosis, the nature of plaque and evaluate the stability of plaque, but also show the situation outside the wall of stenosed artery (8, 9). It has become a noninvasive examination tool for patients with suspected carotid atherosclerosis stenosis. In recent years, with the continuous development of medical imaging technology, people gradually found that the length, thickness, ulcer, texture and other characteristics of carotid plaque are also essentially related to the occurrence of ischemic stroke (8, 10–12). A study by Zaccagna et al. (13) showed that plaque composition and biomechanics are key factors in risk assessment of carotid plaque.

Radiomics is a new research field, which refers to the calculation process of extracting and analyzing a large number of imaging features from medical images. As a computer-aided analysis method of medical images, it can effectively improve the accuracy of detection and classification (14). Compared with traditional assessment, radiomics provides more comprehensive information for disease assessment by converting images into higher-dimensional data and allowing high-through-put extraction of quantitative imaging features (15–17). Radiomics has been widely used in the diagnosis, staging, grading and prognosis evaluation of tumors, and has shown satisfactory results (18–20). In recent years, this technique has also been applied in the quantitative study of carotid and coronary atherosclerotic plaques (15). The researches of Kolossváry et al. (21, 22) showed that radiomics analysis and machine learning based on CTA images can accurately identify high-risk coronary atherosclerotic plaques. Zhang et al. (23) used radiomic features and machine learning to establish a MRI-based high-risk plaque model to distinguish symptomatic and asymptomatic carotid plaques. The research results showed that the MRI-based radiologic model can accurately distinguish symptomatic and asymptomatic carotid plaques. Previous studies have shown that the CTA-based radiomics model can effectively identify symptomatic carotid plaques, and the diagnostic efficiency is significantly better than the routine evaluation of plaques (13, 24–27).

However, most of the current studies only focused on the diagnostic value of a single type of features (such as clinical features, conventional imaging features or radiomic features), but failed to effectively combine radiomic features with traditional risk factors to conduct a more comprehensive and accurate analysis of vulnerable plaques. And most studies only paid attention to the carotid plaques that cause moderate to severe stenosis, but ignored the additional effective information that may be brought by the plaques that cause mild stenosis. Therefore, the purpose of this study is to construct a model that can predict whether carotid plaque will trigger ipsilateral cerebral vascular ischemic events by using the clinical characteristics, plaque imaging features and CTA-based radiomic features, and to verify the reliability of the models through multi-centers.

## 2 Materials and methods

### 2.1 Participants

All the research procedures were approved by the hospital ethics committee (No. ethics (research) No. 012 in 2025), and because this is a retrospective study, the requirement for patients’ written informed consent was waived. Patients who underwent head and neck CTA examination for suspected carotid atherosclerosis disease from August 2022 to July 2025 were screened. The inclusion criteria were as follows: (1) age ≥18 years old; (2) CTA images showed extracranial carotid atherosclerosis; (3) complete clinical data. The exclusion criteria were as follows: (1) insufficient clinical data; (2) history of cerebral hemorrhage, brain tumor, brain trauma or previous brain surgery; (3) history of carotid stenting or carotid endarterectomy; (4) cardiogenic embolism; (5) carotid artery occlusion; (6) CTA images with poor quality; (7) intracranial vascular diseases (such as severe stenosis or occlusion of intracranial atherosclerosis, aneurysm or moyamoya disease, etc.). Finally, according to the above criteria, a total of 223 patients were included, and the demographic, clinical and laboratory data of all patients were collected. The specific status of missing data for clinical variables can be found in Supplementary Table S1.

Patients were divided into symptomatic and asymptomatic group according to neurological evaluation. Patients who experienced ischemic stroke or transient ischemic attack (TIA) in ipsilateral carotid artery territory within the previous 6 months were defined as symptomatic (28). Stroke was defined as focal cerebral or retinal ischemia lasting ≥24 h or resulting in permanent neurological dysfunction (29). TIA was defined as transient neurological dysfunction caused by focal cerebral or retinal ischemia, with symptoms and signs resolving within 24 h (29, 30). Patients were considered asymptomatic if no ipsilateral ischemic events had occurred within the previous 6 months (25).

### 2.2 CTA image acquisition and preprocessing

In Center 1, head and neck CTA was performed using a 256-row CT scanner (Revolution CT, GE Medical System). The patients were supine and scanned from the foot side to the head side. The scanning range was 2 ~ 3 cm below the aortic arch to the cranial top. Injected 50 mL of nonionic iodine contrast agent (iodixanol 320 mgI/ml) through the anterior elbow vein with a double-barrel high-pressure syringe, followed by 50 mL of saline flush, the total injection amount was about 80 ~ 95 mL, and the injection flow rate was 5 mL/s. After injection, monitoring was started with a delay of 2 s, when the contrast agent reached the peak concentration in the target vessel, it began to scan and automatically excited. The monitoring level was set at the descending aorta 2 ~ 3 cm below the aortic arch, and the threshold was 150HU.

The head and neck CTA parameters were as follows: noise index (NI) of 18, rotation speed of 0.5 s, tube voltage of 100 kV, tube current of 100 ~ 500mAs, detector width of 40 mm, slice thickness of 0.625 mm, slice spacing of 0.625 mm, pitch of 0.984: 1, and matrix of 512 × 512.

Scanners, scanning methods and parameters of center 2 and 3 can be found in Supplementary files.

### 2.3 CTA image analysis

The head and neck CTA images of all patients were transmitted to the post-processing workstation (ADW4.6) of the hospital, and the carotid arteries were automatically reconstructed, and the conventional imaging features of carotid plaque were evaluated on the images, including carotid plaque location (common carotid artery, bifurcation of arteria carotis communis and extracranial segment of internal carotid artery), carotid stenosis degree, minimum lumen area, plaque length, maximum plaque thickness, soft plaque thickness, calcification, plaque ulcer, Napkin-Ring Sign (NRS), positive remodeling (PR), common carotid tortuosity index (CCTI) and internal carotid tortuosity index (ICTI). Among them, the degree of carotid stenosis was determined according to CTA standard of North American Symptomatic Carotid Endarterectomy Test (NASCET) (31). CCTI and ICTI are calculated according to the following formula: ([actual distance/linear distance]−1) × 100 (32, 33).

### 2.4 Image segmentation and feature extraction

Before image segmentation, all images were preprocessed through image resampling, gray-level discretization and normalization of window width and window level. Afterwards, all the radiomics analysis were conducted using the Darwin Intelligent Research Platform (Yizhun Medical AI Technology, Beijing, China, https://arxiv.org/abs/2009.00908), including Region of Interest (ROI) labeling, feature extraction and model development. The carotid plaques were segmented on the head and neck CTA axial images. If there were multiple plaques in the same carotid artery, the plaque at the narrowest lumen was selected for segmentation. In the symptomatic group, the carotid plaque on the same side with cerebral ischemia symptoms was selected for segmentation, while in the asymptomatic group, for patients with both carotid plaques, the unilateral plaque with larger volume was selected for segmentation. For patients with both internal and external carotid artery plaques, only the internal carotid artery plaques were segmented (24). Image segmentation was performed by a radiologist with 3 years’ experience in the diagnosis of cervical vascular diseases, and ROI was evaluated by a radiologist with 15 years’ experience in the diagnosis of cervical vascular diseases. Subsequently, a subgroup of patients (*n* = 30) was randomly selected from the study population, and the images were re-segmented by the two radiologists, respectively, 2 weeks later.

Then, radiomic features are extracted, including first-order statistical features, shape features, gray-level co-occurrence matrix (GLCM), gray-level run-length matrix (GLRLM), gray-level size zone matrix (GLSZM), neighboring gray tone difference matrix (NGTDM), gray-level dependence matrix (GLDM) and wavelet features.

### 2.5 Radiomic feature selection

First, the intraclass correlation coefficient (ICC) was calculated for all features, and those with ICC values greater than 0.75 were retained. The retained features were then standardized using min-max normalization (MMN) to ensure comparability. Next, to reduce over-fitting, the top 10% features with the most significant correlation were selected by using Optimal Feature Screening (percentage). Finally, the Least Absolute Shrinkage and Selection Operator (LASSO) algorithm was used to reduce the dimension of features, further selected stable and non-redundant features. The optimal regularization parameter was determined through fivefold cross-validation, and the final set of optimal radiomic features was obtained.

### 2.6 Model construction and verification

Patients from Center 1 were randomly divided into training set and internal test set at the ratio of 8: 2 to ensure the consistency of data distribution and minimize the deviation in data processing. Patients from centers 2 and 3 were included in the external validation set. Based on clinical and imaging features showing statistically significant differences in univariate and multivariate logistic regression analysis, together with the selected optimal radiomic features, three predictive models – the traditional model, radiomic model, and combined model – were constructed using logistic regression to predict ipsilateral cerebral ischemic events associated with carotid plaques.

**Figure 1:**
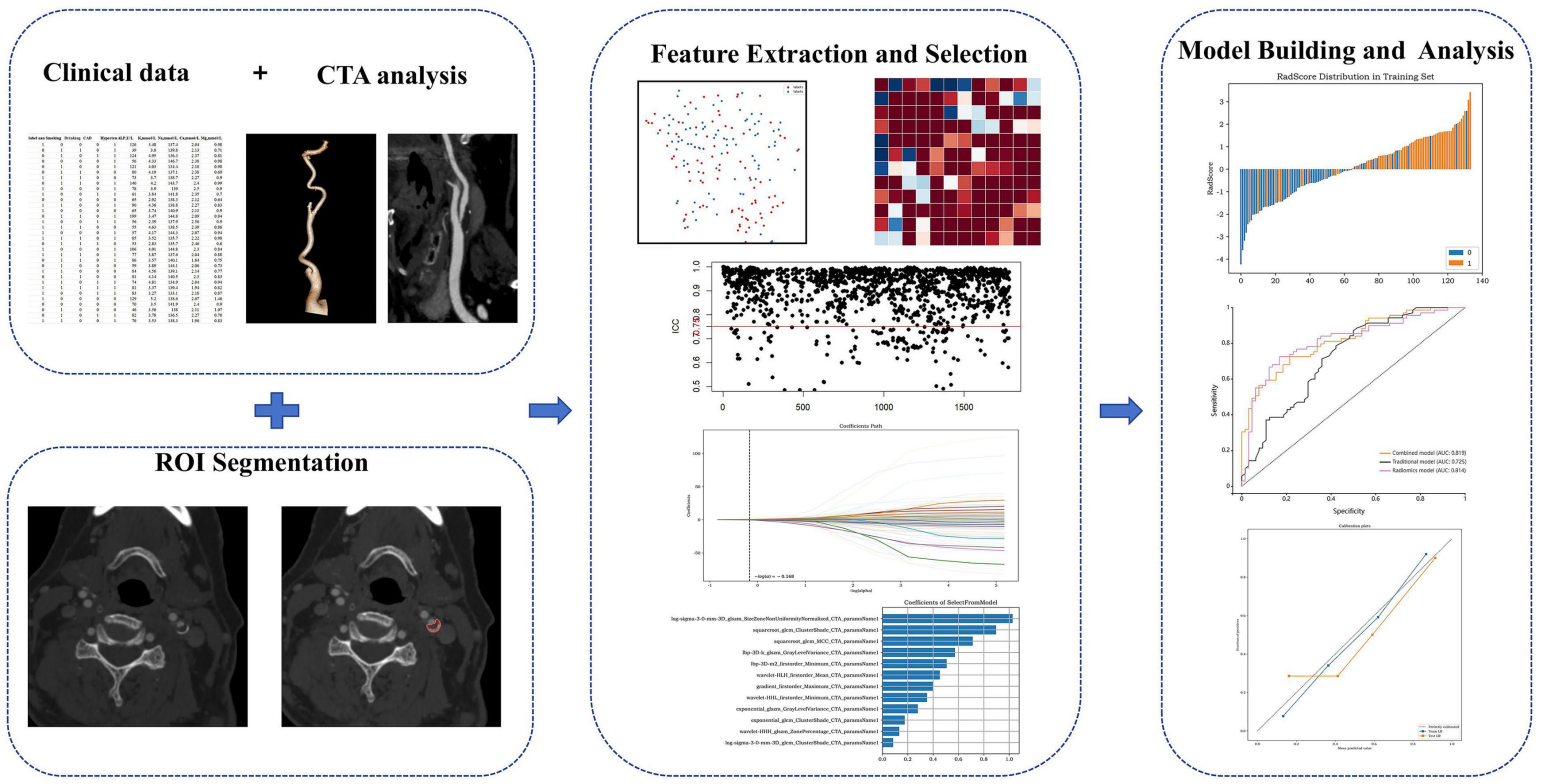
The overall workflow of the radiomics model development. CTA, Computed Tomography Angiography.

The performance of the models was first evaluated in the internal test set, then verified by five-fold cross-validation, and finally further verified in the independent external verification set. Figure 1 shows the workflow of this research.

### 2.7 Statistical analysis

All statistical analyses were performed using SPSS (version 27.0), MedCalc (version 23.3.7) and R software (version 4.5.1). The normality of continuous variables was assessed using the Kolmogorov–Smirnov test. Continuous variables were described as mean ± standard deviation (SD), and categorical variables were expressed as percentages. Difference between the symptomatic and asymptomatic group were evaluated, using the independent-sample t-test or Mann–Whitney *U* test for continuous variables, and the Pearson Chi-square test or Wilcoxon Rank Sum test for categorical variables. Variables with statistical significance in univariate analysis were entered into multivariate logistic regression analysis. The performance of the three predictive models was assessed using receiver operating characteristic curve (ROC) analysis, area under the curve (AUC), sensitivity, specificity, accuracy, calibration curve and decision curve analysis. The Delong test was applied to compare differences in AUCs among the three models. A *p* < 0.05 was considered statistically significant.

## 3 Results

### 3.1 Clinical characteristics

A total of 223 patients were included in the final analysis, including 110 symptomatic patients (93 males and 17 females; Average age, 74.1 ± 8.1 years) and 113 asymptomatic patients (92 males and 21 females; Average age, 72.6 ± 8.7 years). One hundred sixty-eight patients from Center 1 were divided into training set (*n* = 134) and internal test set (*n* = 34), and 55 patients from Centers 2 and 3 were classified as external verification set. The clinical features of all patients are summarized in Table 1. The taking rate of antiplatelet drugs in symptomatic group was higher than that in asymptomatic group (27.27% vs. 15.93%, *p* = 0.040). High-density lipoprotein cholesterol (HDL-C) (*p* = 0.005) and platelet distribution width (PDW) (*p* = 0.006) were significantly different between symptomatic group and asymptomatic group (*p* < 0.05). Multivariate logistic regression analysis showed that PDW (OR = 0.88; 95% CI, 0.80–0.97) is an independent predictor of symptomatic plaque (Table 2).

**Table 1:** Clinical characteristics of patients.

Variables	Total *n* = 223	Symptomatic *n* = 110	Asymptomatic *n* = 113	*p-*value
Age, years	73.327 ± 8.465	74.082 ± 8.143	72.593 ± 8.74	0.189
Sex, male	185(82.96)	93 (84.55)	92 (81.42)	0.535
Smoking	116 (52.02)	60 (54.55)	56 (49.56)	0.457
Drinking	67 (30.04)	30 (27.27)	37 (32.74)	0.374
CAD	45 (20.18)	19 (17.27)	26 (23.01)	0.287
Hypertension	148 (66.37)	79 (71.82)	69 (61.06)	0.090
Diabetes mellitus	65 (29.148)	35 (31.82)	30 (26.55)	0.388
Hyperlipidemia	55 (24.66)	30 (27.27)	25 (22.12)	0.374
Hyperuricemia	15 (6.73)	9 (8.18)	6 (5.31)	0.393
Antihypertension	131 (58.74)	68 (61.82)	63 (55.75)	0.359
Statin	60 (26.91)	33 (30.00)	27 (23.89)	0.305
Antiplatelet	48 (21.52)	30 (27.27)	18 (15.93)	0.040
Antihyperglycemia	55 (24.66)	29 (26.36)	26 (23.01)	0.562
Anticoagulant	17 (7.62)	12 (10.91)	5 (4.42)	0.069
ALP, U/L	83.70 ± 29.81	83.47 ± 25.46	83.92 ± 33.63	0.675
K, mmol/L	4.01 ± 0.61	3.99 ± 0.57	4.03 ± 0.65	0.672
Na, mmol/L	139.09 ± 3.24	138.81 ± 2.74	139.36 ± 3.65	0.202
Ca, mmol/L	2.23 ± 0.20	2.23 ± 0.22	2.24 ± 0.18	0.674
Mg, mmol/L	0.87 ± 0.12	0.89 ± 0.11	0.86 ± 0.11	0.083
P, mmol/L	1.00 ± 0.20	0.98 ± 0.21	1.02 ± 0.19	0.095
Cl, mmol/L	104.10 ± 3.65	104.19 ± 3.46	104.01 ± 3.84	0.723
TG, mmol/L	1.73 ± 1.14	1.67 ± 0.98	1.79 ± 1.28	0.731
TC, mmol/L	4.51 ± 1.20	4.37 ± 1.25	4.64 ± 1.14	0.087
HDL-C, mmol/L	1.21 ± 0.34	1.15 ± 0.27	1.27 ± 0.34	0.005
LDL-C, mmol/L	2.67 ± 0.95	2.58 ± 0.98	2.76 ± 0.92	0.161
Hcy, μmol/L	14.87 ± 5.76	15.43 ± 5.96	14.31 ± 5.52	0.486
PT, sec	11.56 ± 1.26	11.59 ± 1.28	11.52 ± 1.25	0.913
INR	0.98 ± 0.10	0.98 ± 0.11	0.98 ± 0.10	0.706
APTT, sec	28.10 ± 5.31	27.99 ± 6.22	28.19 ± 4.27	0.788
TT, sec	16.76 ± 2.06	16.74 ± 2.23	16.79 ± 1.78	0.617
D-Dimer, mg/L	0.45 ± 0.29	0.48 ± 0.33	0.42 ± 0.26	0.246
BG, mmol/L	7.43 ± 3.21	7.48 ± 2.85	7.37 ± 3.53	0.182
TLC, 10^9^/L	1.5 ± 0.68	1.57 ± 0.76	1.43 ± 0.58	0.484
Hb, g/L	132.09 ± 19.18	132.42 ± 20.72	131.77 ± 17.64	0.670
HCT, %	39.51 ± 6.81	39.83 ± 6.81	39.19 ± 6.82	0.512
RDW-CV, %	14.08 ± 3.73	14.32 ± 3.99	13.85 ± 3.46	0.353
PLT, 10^9^/L	189.14 ± 69.32	194.69 ± 65.28	183.74 ± 72.93	0.111
PDW, fL	14.66 ± 3.15	14.04 ± 2.72	15.27 ± 3.43	0.006
UA, μmol/L	356.93 ± 98.48	363.73 ± 98.85	350.32 ± 98.10	0.310
Scr, μmol/L	89.23 ± 37.45	91.99 ± 36.59	86.53 ± 38.24	0.072

Continuous variables are described as mean ± standard deviation (SD), and categorical variables are presented as *n* (%). ALP, alkaline phosphatase; APTT, activated partial thromboplastin time; BG, blood glucose; CAD, coronary artery disease; HDL-C, high-density lipoprotein cholesterol; Hcy, homocysteine; Hb, hemoglobin; HCT, hematocrit; INR, international normalized ratio; LDL-C, low-density lipoprotein cholesterol; PT, prothrombin time; PLT, blood platelet; RDW-CV, red cell distribution width-coefficient of variation; Scr, Serum creatinine; TG, triglycerides; TC, total cholesterol; TT, thrombin time; TLC, total lymphocyte count; UA, uric acid.

Indicated *p* < 0.05 with significance.

**Table 2:** Multivariable analysis of conventional factors.

Variables	OR 95% CI	*p-*value
Antiplatelet	1.49 (0.71–3.10)	0.288
HDL-C	0.40 (0.14–1.11)	0.079
PDW	0.88 (0.80–0.97)	0.012
Carotid minimum lumen area	0.37 (0.04–3.38)	0.380
Carotid plaque ulceration	5.67 (2.86–11.23)	<0.001
NRS	1.24 (0.66–2.33)	0.510

CI, confidence interval; HDL-C, high-density lipoprotein cholesterol; NRS, napkin ring sign; OR, odds ratio; PDW, platelet distribution width.

### 3.2 Plaque imaging features on CTA image

Univariate analysis showed that plaque ulcer (*p* < 0.001), minimum lumen area (*p* = 0.001) and napkin ring sign (*p* = 0.036) were related to plaque vulnerability (Table 3). Further multivariate logistic regression analysis showed that plaque ulcer (OR = 5.67; 95% CI, 2.86–11.23) is an independent predictor of symptomatic plaque (Table 2).

**Table 3:** Imaging features of patients.

Variables		Total *n* = 223	Symptomatic *n* = 110	Asymptomatic *n* = 113	*p-*value
Plaque site	0	40 (17.94)	18 (16.36)	22 (19.47)	0.374
	1	41 (18.39)	19 (17.27)	22 (19.47)	
	2	142 (63.68)	73 (66.36)	69 (61.06)	
Degree of stenosis	0	22 (9.87)	13 (11.82)	9 (7.96)	0.126
	1	118 (52.91)	48 (43.64)	70 (61.95)	
	2	83 (37.22)	49 (44.55)	34 (30.09)	
Carotid minimum lumen area, cm^3^		0.18 ± 0.14	0.16 ± 0.15	0.20 ± 0.13	0.001
Carotid plaque length, mm		18.37 ± 9.66	18.19 ± 9.01	18.54 ± 10.30	0.814
Carotid maximum total plaque thickness, mm		4.50 ± 1.367	4.42 ± 1.35	4.58 ± 1.38	0.391
Maximum soft plaque thickness, mm		3.44 ± 1.71	3.62 ± 1.62	3.27 ± 1.78	0.072
Plaque calcification		192 (86.09)	96 (87.27)	96 (84.95)	0.618
Carotid plaque ulceration		71 (31.84)	55 (50.00)	16 (14.16)	<0.001
NRS		82 (36.77)	48 (43.64)	34 (30.10)	0.036
PR		96 (43.05)	52 (47.27)	44 (38.94)	0.210
CCTI, %		5.27 ± 4.42	5.07 ± 4.26	5.46 ± 4.58	0.749
ICTI, %		13.61 ± 9.57	13.53 ± 9.60	13.69 ± 9.59	0.949

Continuous variables are described as mean ± standard deviation (SD), and categorical variables are presented as numbers (%). Plaque site: 0 (arteria carotis communis), 1 (Bifurcation of arteria carotis communis), 2 (internal carotid). Degree of stenosis: 0 (0–49.9%), 1 (50–69.9%), 2 (70–99.9%). CCTI, common carotid tortuosity index; ICTI, internal carotid tortuosity index; NRS, napkin ring sign; PR, positive remodeling.

### 3.3 Radiomic analysis of plaque

**Figure 2:**
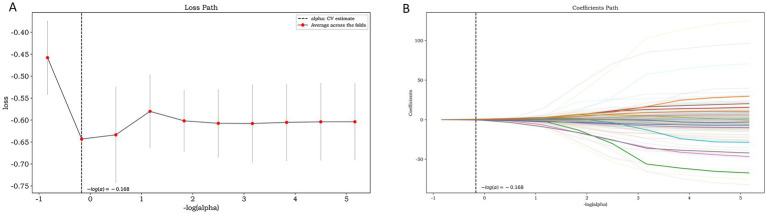
Feature selection using LASSO regression: the loss path of LASSO **(A)** and the regression coefficients of LASSO **(B)**.

**Figure 3:**
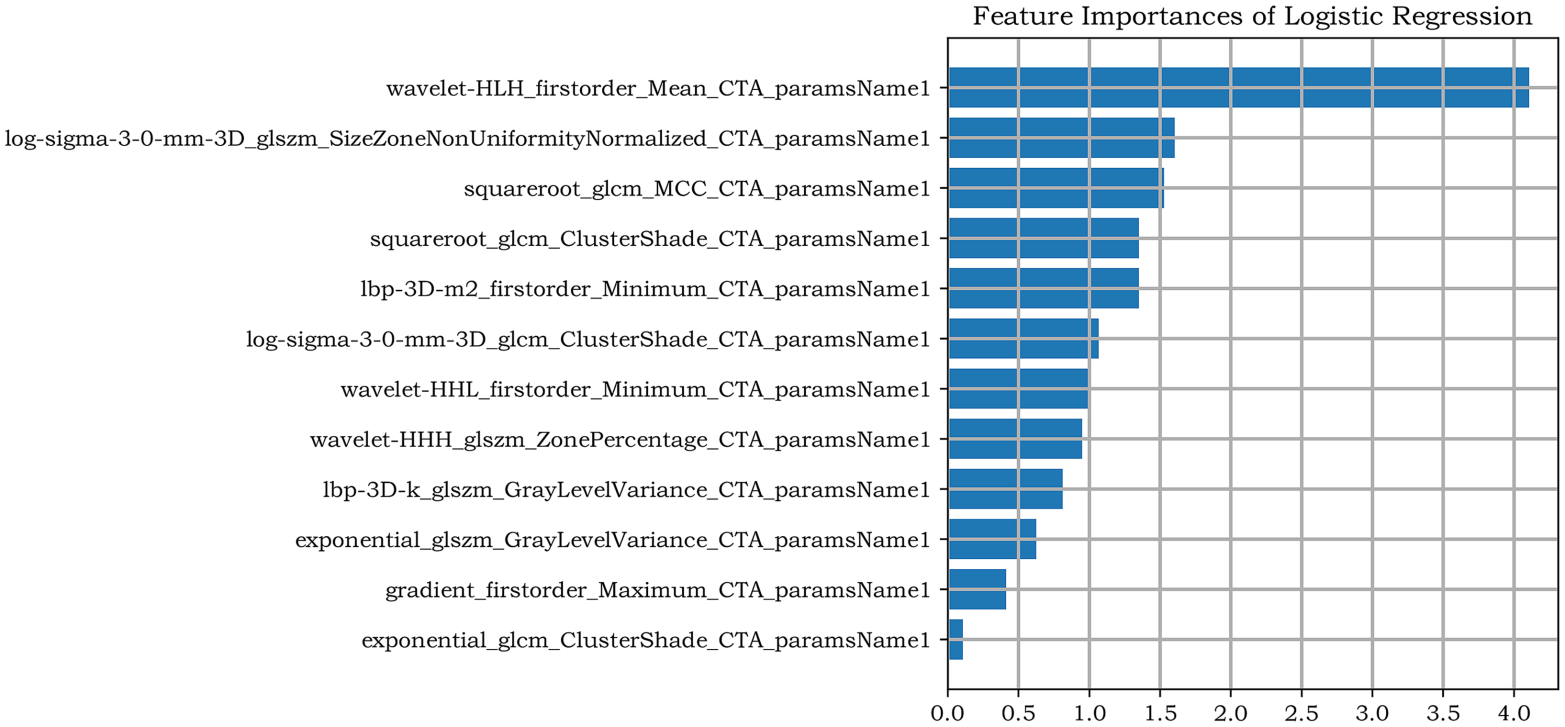
The remaining features of LASSO regression screening and the ranking of weight coefficients output by the corresponding LR classifier under the Radiomics model.

A total of 1781 features were extracted, and the radiomic features with ICC > 0.75 were retained (The ICC calculation results of intra-observer consistency and inter-observer consistency can be found in Supplementary Figure S1). Finally, a total of 1,442 stable features were included for subsequent analysis, including 175 first-order statistical features, 12 shape features, 180 GLCM features, 146 GLRLM features, 122 GLSZM features, 38 NGTDM features, 132 GLDM features and 637 wavelet features. The logistic regression algorithm provided by the Darwin Intelligent Research Platform is adopted, and 12 reserved optimal radiomic features (Figure 2 illustrates the feature selection using LASSO regression) were used to construct the radiomics model. To facilitate clinical understanding and biological interpretation, these features are detailed in Supplementary Table S2, which includes their category, a IBSI-compliant definition, and a contextual interpretation specific to carotid plaque vulnerability. These 12 features and their importance in the logistic regression model are shown in Figure 3.

### 3.4 Model construction and performance evaluation

**Figure 4:**
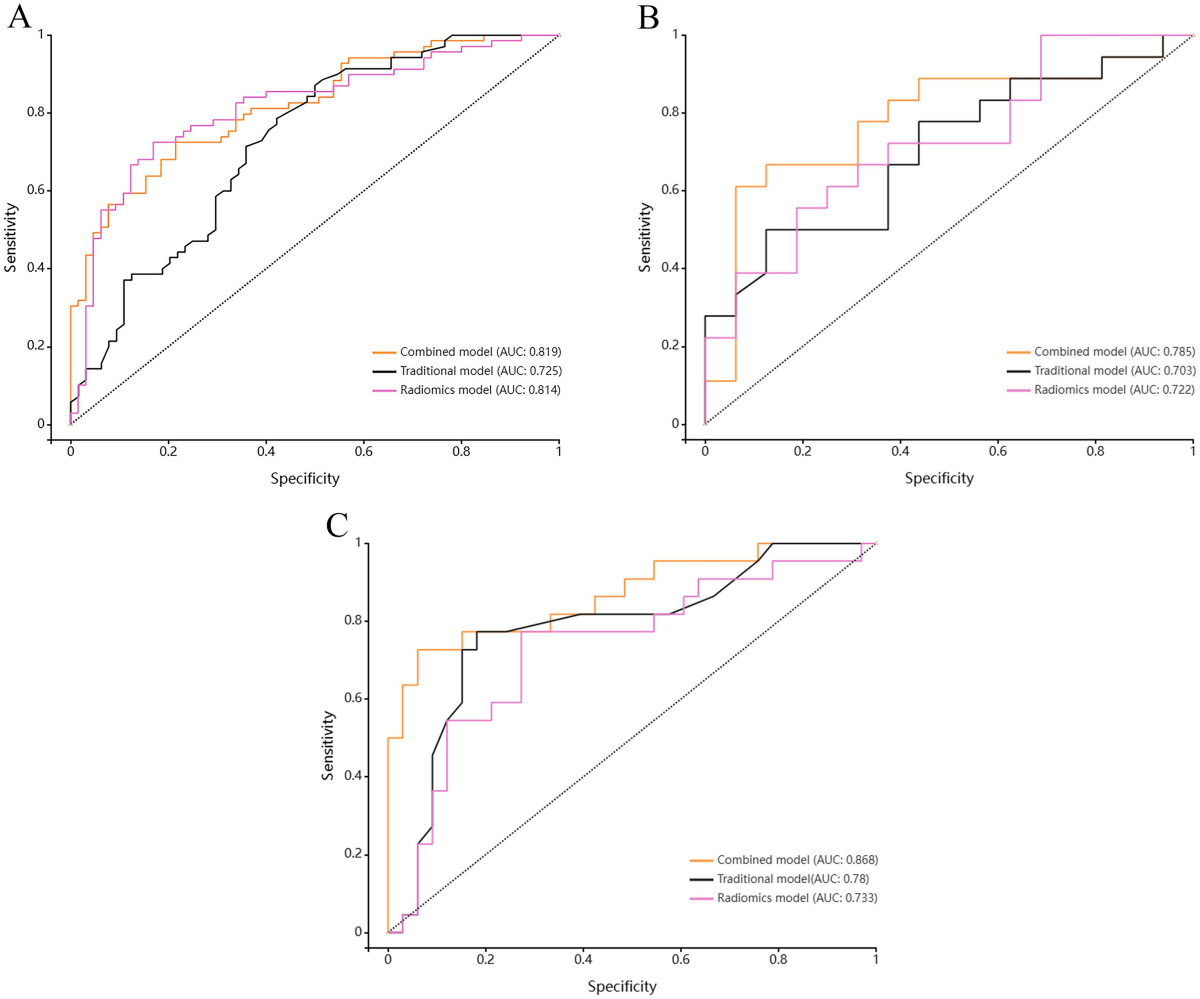
Comparison of ROC curves among the Radiomics model, the Traditional model, and the Combined model for **(A)** the training set, **(B)** the test set, and **(C)** the validation set.

**Figure 5:**
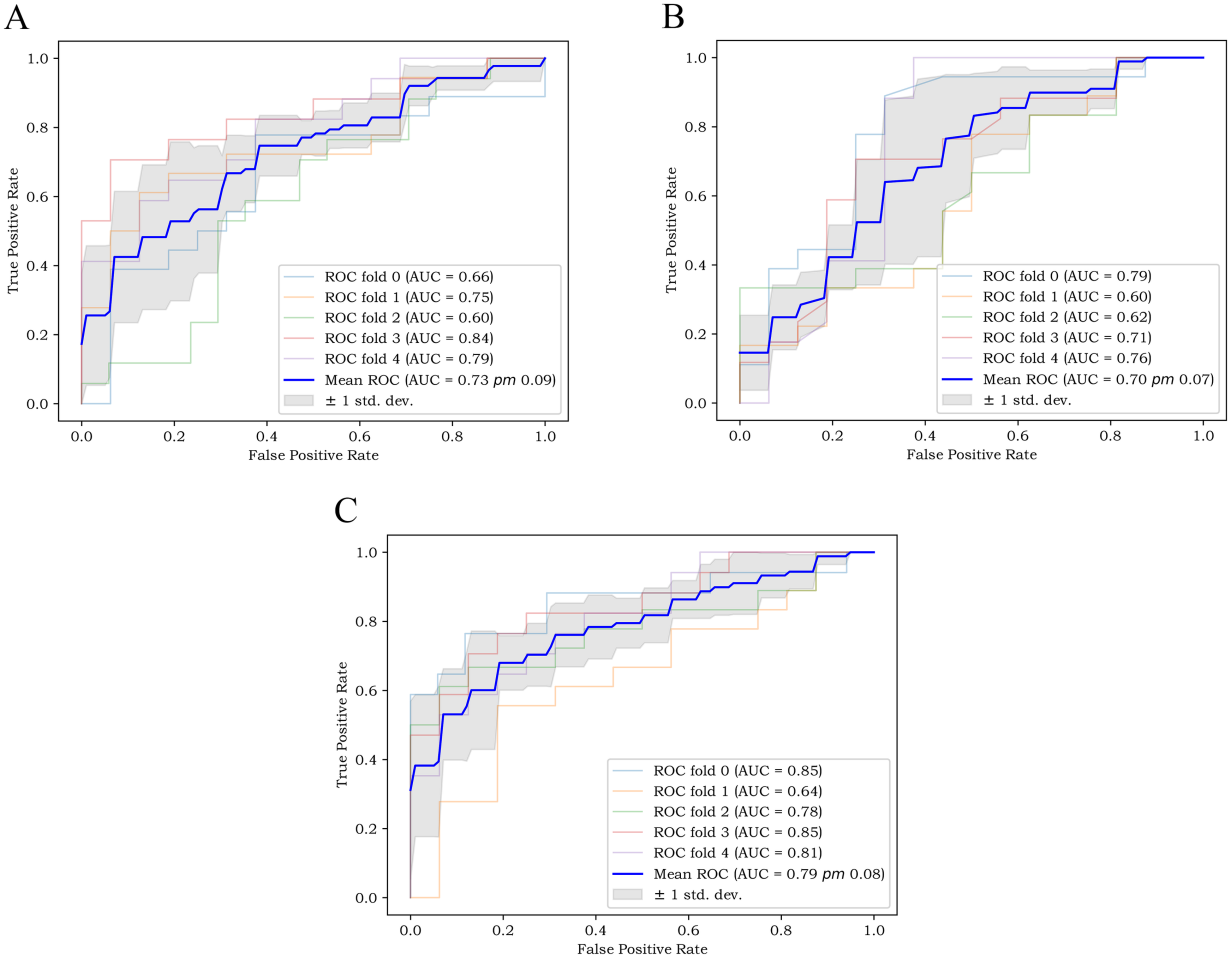
The five-fold cross-validation ROC curves of **(A)** the radiomics model, **(B)** the traditional model, and **(C)** the combined model.

Based on the above-mentioned two independent related factors and 12 significant radiomic features, we established the traditional model, radiomics model and combined model. The hyperparameter settings of three models can be found in Supplementary files. Table 4 summarizes the diagnostic performance of each model. ROC curves of the training set, internal test set and external verification set of the three models are shown in Figure 4. The five-fold cross-validation ROC curves of the three models are shown in Figure 5.

**Table 4:** Diagnostic performance of three models for identification of symptomatic plaque.

Cohorts	Assessment models	AUC (95%CI)	SEN	SPE	PPV	NPV	ACC
Training set	Radiomics model	0.814 (0.740–0.889)	0.725	0.831	0.820	0.740	0.776
	Traditional model	0.725 (0.639–0.812)	0.729	0.609	0.671	0.672	0.672
	Combined model	0.819 (0.749–0.888)	0.725	0.769	0.769	0.725	0.746
Test set	Radiomics model	0.722 (0.549–0.896)	0.667	0.625	0.667	0.625	0.647
	Traditional model	0.703 (0.525–0.881)	0.500	0.813	0.750	0.591	0.647
	Combined model	0.785 (0.620–0.950)	0.667	0.813	0.800	0.684	0.735
Validation set	Radiomics model	0.733 (0.591–0.875)	0.773	0.727	0.654	0.828	0.745
	Traditional model	0.780 (0.648–0.913)	0.773	0.818	0.739	0.844	0.800
	Combined model	0.868 (0.765–0.970)	0.727	0.939	0.889	0.838	0.855

AUC, area under the curve; ACC, accuracy; NPV, negative predictive value; PPV, positive predictive value; SEN, sensitivity; SPE, specificity.

The radiomics model showed favorable diagnostic performance. For training set, the AUC was 0.814 (95% CI: 0.740–0.889), the sensitivity was 72.5%, the specificity was 83.1%, and the accuracy was 77.6%. For internal test set the AUC was 0.722 (95% CI: 0.549–0.896), the sensitivity was 66.7%, the specificity was 62.5%, and the accuracy was 64.7%. And for external validation set, the AUC was 0.733 (95% CI: 0.591–0.875), the sensitivity was 77.3%, the specificity was 72.7%, and the accuracy was 74.5%.

The diagnostic performance of traditional model for symptomatic plaques was relatively low. For training set, the AUC was 0.725 (95% CI: 0.639–0.812), the sensitivity was 72.9%, the specificity was 60.9%, and the accuracy was 67.2%. For internal test set the AUC was 0.703 (95% CI: 0.525–0.881), the sensitivity was 50.0%, the specificity was 81.3%, and the accuracy was 64.7%. And for external validation set, the AUC was 0.780 (95% CI: 0.648–0.913), the sensitivity was 77.3%, the specificity was 81.8%, and the accuracy was 80.0%.

The diagnostic performance of combined model was higher than that of radiomics model and traditional model. For training set, the AUC was 0.819 (95% CI: 0.749–0.888), the sensitivity was 72.5%, the specificity was 76.9%, and the accuracy was 74.6%. For internal test set the AUC was 0.785 (95% CI: 0.620–0.950), the sensitivity was 66.7%, the specificity was 81.3%, and the accuracy was 73.5%. And for external validation set, the AUC was 0.868 (95% CI: 0.765–0.970), the sensitivity was 72.7%, the specificity was 93.9%, and the accuracy was 85.5%.

The five-fold cross-validation results of the three models showed that the mean AUC values of the radiomics model, the traditional model and the combined model were 0.73, 0.70 and 0.79, respectively.

**Figure 6:**
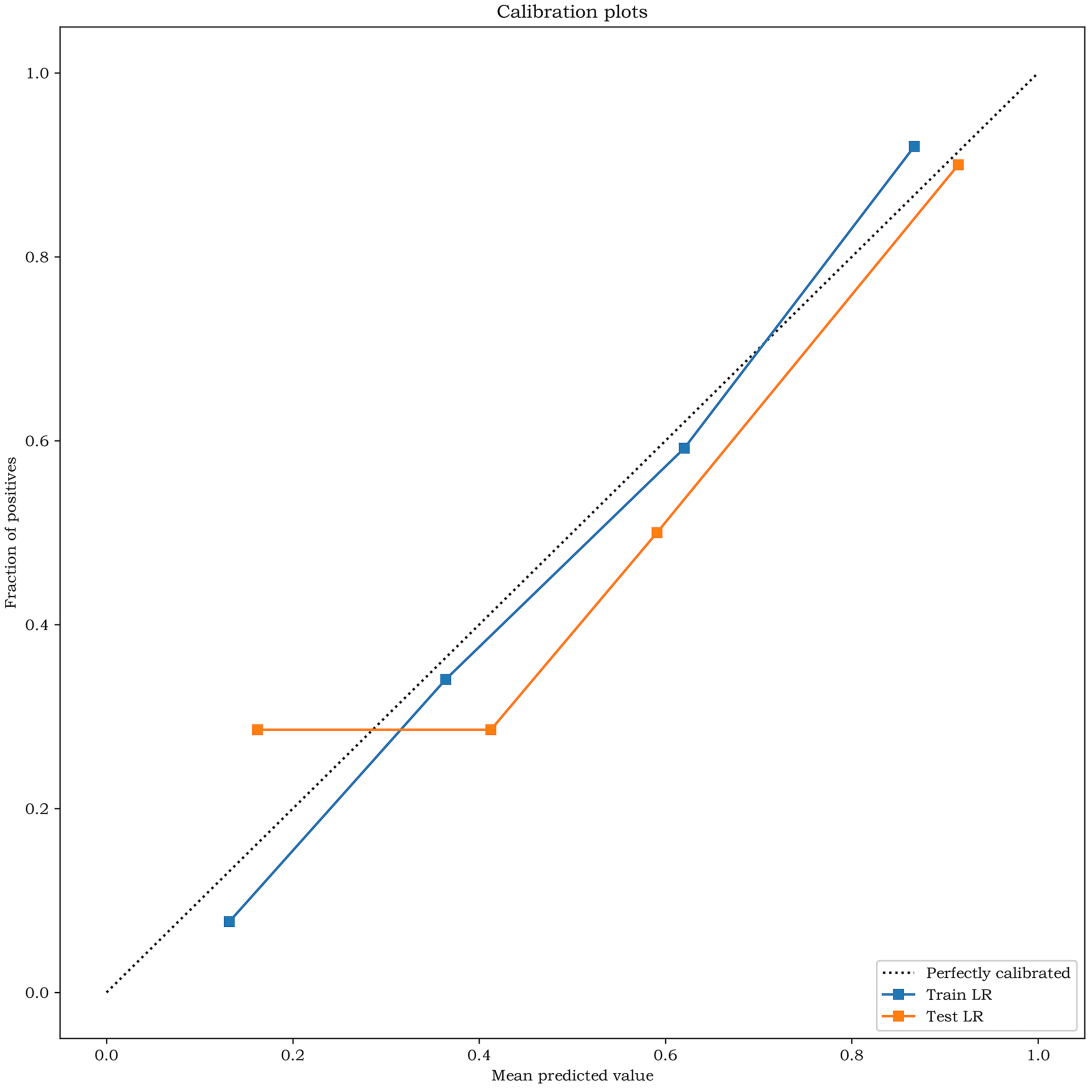
The calibration curves analysis for the training set and the test set of the combined model. The closer the calibration curve is to the dotted line, the higher the calibration degree of the model.

**Figure 7:**
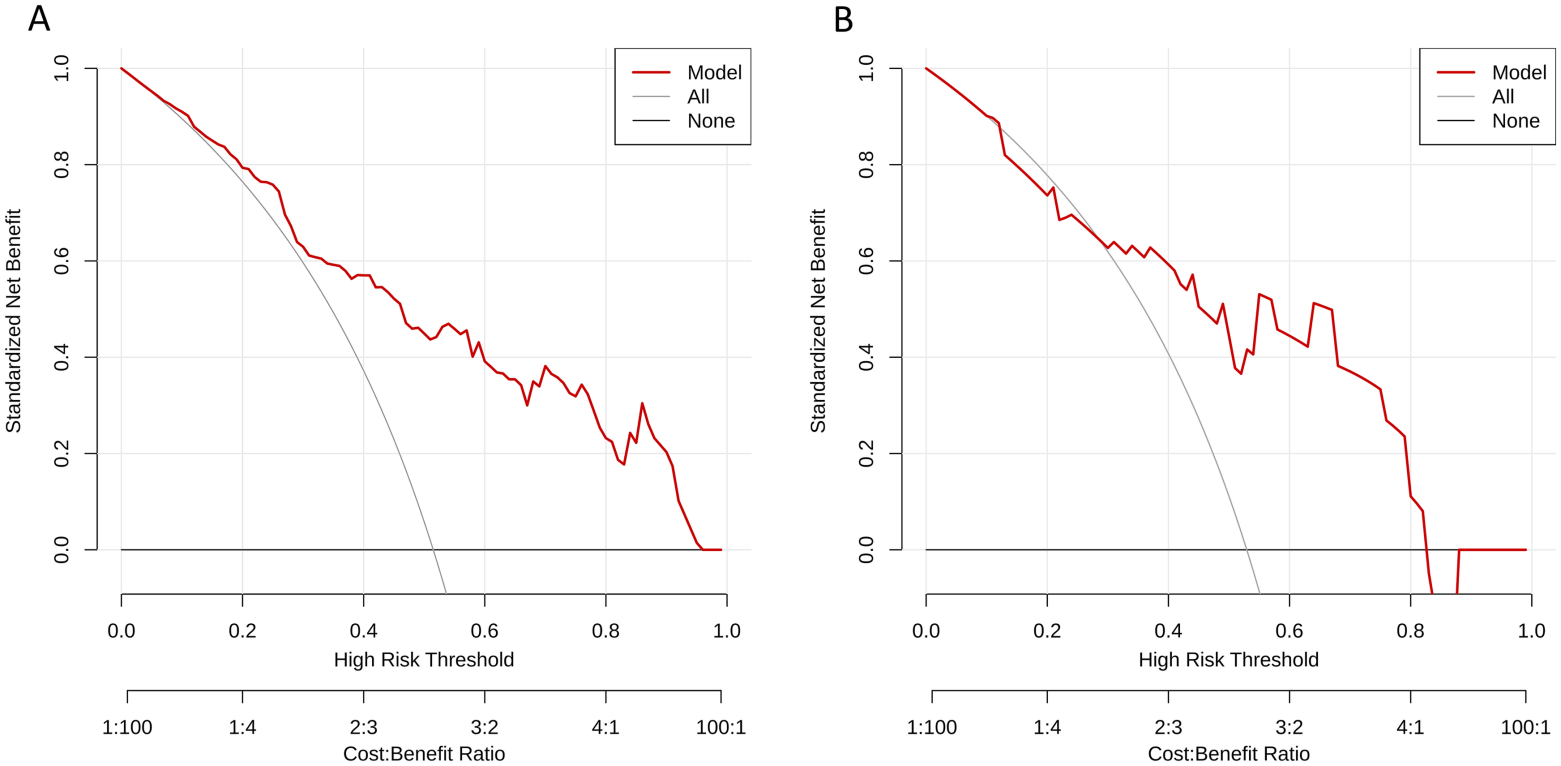
Decision curves analysis for **(A)** the training set and **(B)** the test set of the combined model. The *y*-axis represents the net benefit; *x*-axis represents threshold probability. The red line indicate, respectively, net benefits of the combined model. The gray line indicates the hypothesis that all patients had symptomatic carotid plaques. The black horizontal line indicates the hypothesis that no patients had symptomatic carotid plaques.

The results of Delong test and Integrated Discrimination Index (IDI) are shown in Table 5. In the external verification set, the diagnostic efficiency of combined model was better than that of radiomics model (AUC 0.868 vs. 0.733, *p* = 0.038). The calibration curves of the combined model proved a good fit between the prediction and the actual possibility of ischemic stroke in the training set and the internal test set (Figure 6). The decision curves showed that the combined model has a good overall net benefit (Figure 7).

**Table 5:** Delong test and integrated discrimination index (IDI) results for the training set, the test set, and the validation set of the three models.

Assessment models	Cohorts	*p*(Delong test)	IDI	*p*(IDI)
Traditional model – Radiomics model	Training set	0.092	0.233	<0.001
	Test set	0.878	0.163	–
	Validation set	0.652	0.105	–
Traditional model – Combined model	Training set	0.002	0.069	<0.001
	Test set	0.284	0.076	0.018
	Validation set	0.093	0.082	<0.001
Radiomics model – Combined model	Training set	0.903	–	–
	Test set	0.399	–	–
	Validation set	0.038	–	–

## 4 Discussion

This study evaluated clinical variables, laboratory indicators, plaque imaging features and radiomic features, and three prediction models (traditional model, radiomics model and combined model) were established by logistic regression algorithm to predict the occurrence of ipsilateral cerebral ischemic events of carotid plaque. The results show that the diagnostic performance of radiomics model and traditional model combining clinical features and plaque imaging features was relatively low, while the combined model combining traditional characteristics and radiomic features could better distinguish symptomatic patients from asymptomatic patients.

Previous studies have shown that high Homocysteine (Hcy) level is an independent risk factor for cerebral ischemic events (24, 27), and patients with Hcy level higher than 15 mol/L have a significantly increased risk of ischemic stroke (34). However, the result of this study showed that there was no significant difference in Hcy value between the symptomatic group and the asymptomatic group (*p* = 0.48), which was inconsistent with previous research results. This result may be caused by factors such as relatively small sample size and limited Hcy value of patients included. This study first proposed the correlation between PDW (OR = 0.88; 95% CI, 0.80–0.97) and plaque vulnerability. PDW is an index reflecting the uniformity of platelet volume and size in peripheral blood, and its high value indicates that the production of larger reticular platelets increases, which is an important index reflecting platelet production and may be indirectly related to platelet function and activity (35). One study suggested that the increase of PDW value may be potentially related to acute ischemic stroke (36). The result of this study showed that PDW was a protective factor of cerebral ischemic events. The overall PDW level of symptomatic group was higher than that of asymptomatic group. However, due to past events, patients in symptomatic group are more likely to receive antiplatelet therapy, so this result was most likely the mixed effect brought by antiplatelet therapy, not the biological effect of PDW itself. Therefore, we divided all patients into two subgroups, namely, the subgroup without antiplatelet therapy and the subgroup with antiplatelet therapy. Multivariate logistic regression analysis was carried out in the two subgroups. The result showed that PDW was still a protective factor of cerebral ischemia events in the subgroup without antiplatelet therapy (OR = 0.86; 95% CI, 0.77–0.96; *p* < 0.001), which indicated that the role of PDW was independent of antiplatelet therapy to some extent. This finding is inconsistent with the previous research results. In the future, we need to use larger datasets or more refined statistical models to verify this result, and more related researches are needed to explore whether there is a specific platelet subtype that responds well to treatment.

Previous studies have shown that the measurement of carotid plaque stenosis can effectively predict the occurrence of ipsilateral ischemic events (37). In recent years, more and more scholars have studied the correlation between carotid atherosclerotic plaque and ischemic cerebrovascular disease. Some studies have found that for patients with mild to moderate carotid stenosis, the increase of stenosis degree is not necessarily related to the higher risk of ischemic stroke (38). Gorgui et al. (39) and Baradaran et al. (11) found that for patients with symptomatic carotid plaque, even with mild stenosis, ulcers, rupture, bleeding and superficial thrombosis can be seen in the plaque, but the degree of inclusion is different, suggesting that only evaluating lumen stenosis will underestimate the risk of carotid plaque. There was some evidence that the occurrence of stroke and TIA is also significantly related to the composition and vulnerability of plaque, not just to the stenosis of carotid atherosclerosis (40–42). Previous researches have emphasized that some conventional imaging features of plaque, such as soft plaque, Intraplaque hemorrhage (IPH), lipid-rich necrotic core (LRNC) and thin fibrous cap, are closely related to plaque instability, and have been supported by pathological research (43). Therefore, more attention should be paid to the comprehensive evaluation of all possible risk factors. Imaging examination plays a vital role in the evaluation of carotid plaque, among which CTA has been widely used in clinical practice. In this study, we measured and analyzed the conventional imaging features of carotid plaque on CTA images. The results of univariate analysis showed that plaque ulcer, minimum lumen area and napkin ring sign were related to plaque vulnerability. Some studies have indicated that the plaque napkin ring sign is a high-risk sign of ischemic stroke (24, 27), which may indicate the dysfunction of adventitia neovascularization and bleeding tendency, which is consistent with our research results. Further multivariate logistic regression analysis showed that plaque ulcer (OR = 5.67; 95% CI, 2.86–11.23) is an independent predictor of symptomatic plaque, which is consistent with previous studies (24, 42, 44). Multiple studies have found that plaque ulcers increase the risk of ipsilateral cerebral ischemia events by 2.2 times (45).

However, the analysis of plaque image characteristics on CTA images is still limited to the representation level. Lambin et al. (46) first put forward radiomics, a new medical image analysis technology, which extracts a large number of subtle features that are difficult for human eyes to detect by segmenting the region of interest in medical images, and then uses computer algorithms to convert these features into higher-dimensional data for further analysis. Over the past 10 years, radiomics has been widely used in oncology related fields and achieved satisfactory results, but its application in cardiovascular and cerebrovascular diseases was relatively backward. In recent years, there were more and more radiomics studies related to cardiovascular and cerebrovascular diseases. Le et al. (47) proved the robustness of CTA-based radiomics in identifying criminal lesions in cerebrovascular events. Radiomics analysis has also made great progress in identifying symptomatic carotid plaque. The research of Zaccagna et al. (13) showed that the analysis of carotid plaque texture characteristics based on CTA images can be used as a new risk stratification method for patients with carotid atherosclerosis. Shi et al. (24) and Liu et al. (25) conducted a radiomics analysis of carotid plaques based on CTA images and established nomograms to identify high-risk carotid plaques, and the results showed that the radiomic features of plaques can effectively evaluate the vulnerability of plaques. The study of Li et al. (27) included 123 patients with carotid atherosclerosis, including 64 symptomatic patients and 59 asymptomatic patients. The ROI was delineated on the single-slice CTA image at the narrowest level of the extracranial carotid canal cavity for radiomics analysis. The result showed that the model combining radiomic features and clinical features could effectively identify symptomatic carotid plaques. Therefore, on the basis of previous studies, this study constructed a traditional model combining clinical risk factors and plaque imaging features and a radiomics model based on CTA images, and established a combined model combining traditional risk factors and plaque radiomic features to explore its application value in predicting ipsilateral cerebral ischemic events caused by carotid plaque.

This study has obvious advantages in the dimensions of data acquisition cost, interpretability, reproducibility, and cross-center transferability. First of all, this study made full use of the “by-product” data in the existing clinical process. All clinical features were from electronic medical records, which were part of routine diagnosis and treatment, and the marginal cost is almost zero. Head and neck CTA has been one of the standard imaging examinations for patients with carotid atherosclerosis, and the radiomics features were extracted from existing CTA images, which increases the “added value” of the data without increasing the patient’s economic and radiation burden. Secondly, all the included clinical features have clear biological and pathophysiological significance, which is easy for patients to understand and trust. Thirdly, this study is a multi-center study, and the results showed that our model has good generalization ability for different scanning devices, protocols and patient groups. In addition, we adopted standardized radiomics processing procedures (such as image standardization and preprocessing, ROI delineation, feature extraction and screening), which greatly improved the repeatability of methodology.

Additionally, compared with previous radiomics studies based on carotid plaque, this study has made several improvements. First of all, this study included patients with carotid atherosclerosis of all stenosis degrees, not only patients with high stenosis (>50%), in order to avoid missing some relevant information as much as possible. Secondly, in this study, the three-dimensional ROI of extracranial carotid plaque was analyzed to obtain more characteristics and heterogeneity than that of the two-dimensional ROI. Thirdly, most of the previous studies were single-center and the sample size was limited, which limited the stability and wide applicability of the model. The multi-center nature of this study significantly increased the generalization of the proposed model by improving the diversity and representativeness of the samples. In this study, 12 optimal radiomic features were finally selected for the development of radiomics model.

The final ROC curve analysis results showed that the combined model has the highest diagnostic efficiency, and the three models show consistent performance in the training set, internal test set and external verification set, which showed that our data set is representative enough and our models have good generalization ability. Delong test showed that the combined model has better diagnostic efficiency than the radiomics model in the external verification set (*p* = 0.038). Although there was no significant difference in the performance between the radiomics model and the traditional model by Delong test, the IDI analysis showed that the performance of the radiomics model was significantly better than that of the traditional model in the training set (IDI value = 0.233, *p* < 0.001). Although Delong test showed that the combined model has no significant advantages compared with the traditional model (*p*-values in training set, test set and external verification set are all > 0.05), IDI analysis showed that the performance of the combined model is significantly better than the traditional model (training set: IDI value = 0.069, *p* < 0.001; internal test set: IDI value = 0.076, *p* = 0.018; external verification set: IDI value = 0.082, *p* < 0.001), which showed that, compared with the single traditional model, the addition of radiomic features makes the combined model show better performance in identifying symptomatic plaques. Generally speaking, the combined model had the best diagnostic efficiency, and it had better predictive ability than the single model.

Despite the satisfactory results, there are still some limitations in this study. First of all, the sample size of this study is relatively small, and in order to provide better evidence for clinical application, a larger sample size may be needed for further verification. Secondly, this study is a retrospective study, and it cannot completely ensure its prediction of subsequent cerebral ischemia events, so prospective studies are needed to evaluate the long-term accuracy and stability. Thirdly, we included patients with extracranial carotid atherosclerosis, although we tried to exclude other possible causes of cerebral ischemic events, we still could not sure that all cerebral ischemic events were caused by carotid atherosclerotic plaques. Fourthly, the analysis of radiomics needs manual segmentation of three-dimensional ROI, which is complex and time-consuming, and there is inevitable subjectivity and variability. In the future, an effective automatic segmentation method is needed to improve efficiency and accuracy, overcome current bottlenecks in radiomics workflows, thereby advancing the widespread clinical application of this technology.

## 5 Conclusion

To sum up, this study showed that the clinical characteristics of patients and the CTA-based imaging features of carotid atherosclerotic plaques, such as PDW and plaque ulcer, have independent predictive value for identifying symptomatic plaques. CTA-based radiomics analysis of carotid atherosclerosis also effectively distinguished symptomatic from asymptomatic plaques. Moreover, the combination of traditional clinical characteristics, imaging features and radiomic features can produce better prediction efficiency, which may help clinicians to conduct more comprehensive and accurate risk assessments for patients with carotid atherosclerosis, identify high-risk individuals, and help low-risk patients avoid unnecessary invasive treatments.

## Data Availability

The raw data supporting the conclusions of this article will be made available by the authors, without undue reservation.
