# Supplementary material for: Identification of symptomatic carotid plaque by CTA-based radiomics: a multicenter study
Source: Front Neurol. 2026 Jan 21;17:1750076. doi: 10.3389/fneur.2026.1750076 (PMC12867918; doi:10.3389/fneur.2026.1750076)
Supplement: Supplementary file 1 [file Supplementary_file_1.docx]

In Center 2, head and neck CTA was performed using a 256-row CT scanner (IQon-Spectral CT, Philips Medical System). The patients were supine and scanned from the foot side to the head side. The scanning range was 2~3cm below the aortic arch to the cranial top. Injected nonionic iodine contrast agent (iodixanol 350mgI/ml) through the median elbow vein with a double-barrel high-pressure syringe, and the dose was calculated according to the body weight (1.5ml/kg for adults), followed by 30ml of saline ﬂush, and the injection flow rate was 5ml/s. After injection, monitoring was started with a delay of 2s, when the contrast agent reached the peak concentration in the target vessel, it began to scan and automatically excited. The monitoring level was set at the descending aorta 2~3cm below the aortic arch, and the threshold was 150HU.

The head and neck CTA parameters were as follows: rotation speed of 0.33s, tube voltage of 120kV, tube current of 182mAs, collimation width of 128mm, slice thickness of 0.9mm, slice spacing of 0.45mm, pitch of 1.015: 1, and matrix of 512×512.

In Center 3, head and neck CTA was performed using a 256-row CT scanner (uCT 968, United Imaging Medical System). The patients were supine and scanned from the head side to the foot side. The scanning range was 2~3cm below the aortic arch to the cranial top. Injected 60ml nonionic iodine contrast agent (iodixanol 350 or 370mgI/ml) through the median elbow vein with a double-barrel high-pressure syringe, and the dose was calculated according to the body weight (1.5ml/kg for adults), followed by 30ml of saline ﬂush, the total injection amount was about 90ml, and the injection flow rate was 5ml/s. After injection, monitoring was started with a delay of 2s, when the contrast agent reached the peak concentration in the target vessel, it began to scan and automatically excited. The monitoring level was set at the descending aorta 2~3cm below the aortic arch, and the threshold was 150HU.

The head and neck CTA parameters were as follows: rotation speed of 0.28s, tube voltage of 100kV, tube current of 220mAs, collimation width of 80mm, slice thickness of 0.5mm, slice spacing of 0.5mm, pitch of 0.9188: 1, and matrix of 512×512.
