# Supplementary material for: Identification of symptomatic carotid plaque by CTA-based radiomics: a multicenter study
Source: Front Neurol. 2026 Jan 21;17:1750076. doi: 10.3389/fneur.2026.1750076 (PMC12867918; doi:10.3389/fneur.2026.1750076)
Supplement: Supplementary file 2 [file Supplementary_file_2.docx]

For the logistic regression models, the penalty was set to elasticnet. The inverse regularization parameter (C) was searched over 0.001, 0.01, 0.1, 1, 10, 100, and 1000, and l1_ratio was explored from 0.1 to 0.9 in steps of 0.1. The random_state was fixed at 2 to ensure reproducibility.

The optimal hyperparameters were selected based on model performance: radiomics model (C = 100, l1_ratio = 0.1), conventional model (C = 0.1, l1_ratio = 0.5), and combined model (C = 0.1, l1_ratio = 0.1).
