# Supplementary material for: Identification of symptomatic carotid plaque by CTA-based radiomics: a multicenter study
Source: Front Neurol. 2026 Jan 21;17:1750076. doi: 10.3389/fneur.2026.1750076 (PMC12867918; doi:10.3389/fneur.2026.1750076)
Supplement: Supplementary file 3 [file Table_1.docx]

| **Variables** | **Missing quantity** | **Missing rate, %** |
| --- | --- | --- |
| Age, years | 0 | 0 |
| Sex, male | 0 | 0 |
| Smoking | 0 | 0 |
| Drinking | 0 | 0 |
| CAD | 0 | 0 |
| Hypertension | 0 | 0 |
| Diabetes mellitus | 0 | 0 |
| Hyperlipidemia | 0 | 0 |
| Hyperuricemia | 0 | 0 |
| Antihypertension | 0 | 0 |
| Statin | 0 | 0 |
| Antiplatelet | 0 | 0 |
| Antihyperglycemia | 0 | 0 |
| Anticoagulant | 0 | 0 |
| ALP, U/L | 21 | 9.4 |
| K, mmol/L | 10 | 4.5 |
| Na, mmol/L | 10 | 4.5 |
| Ca, mmol/L | 12 | 5.4 |
| Mg, mmol/L | 13 | 5.8 |
| P, mmol/L | 9 | 4.0 |
| Cl, mmol/L | 10 | 4.5 |
| TG, mmol/L | 11 | 4.9 |
| TC, mmol/L | 11 | 4.9 |
| HDL-C, mmol/L | 11 | 4.9 |
| LDL-C, mmol/L | 15 | 6.7 |
| Hcy, μmol/L | 17 | 7.6 |
| PT, sec | 15 | 6.7 |
| INR | 13 | 5.8 |
| APTT, sec | 15 | 6.7 |
| TT, sec | 17 | 7.6 |
| D-Dimer, mg/L | 18 | 8.1 |
| BG, mmol/L | 0 | 0 |
| TLC, 10^9^/L | 11 | 4.9 |
| Hb, g/L | 11 | 4.9 |
| HCT, % | 11 | 4.9 |
| RDW-CV, % | 10 | 4.5 |
| PLT, 10^9^/L | 10 | 4.5 |
| PDW,fL | 13 | 5.8 |
| UA, μmol/L | 20 | 8.9 |
| Scr, μmol/L | 16 | 7.2 |

ALP, alkaline phosphatase; APTT, activated partial thromboplastin time; BG, blood glucose; CAD, coronary artery disease; HDL-C, high-density lipoprotein cholesterol; Hcy, homocysteine; Hb, hemoglobin; HCT, hematocrit; INR, international normalized ratio; LDL-C, low-density lipoprotein cholesterol; PT, prothrombin time; PLT, blood platelet; RDW-CV, red cell distribution width-coefficient of variation; Scr, Serum creatinine; TG, triglycerides; TC, total cholesterol; TT, thrombin time; TLC, total lymphocyte count; UA, uric acid.
