# Supplementary material for: Identification of symptomatic carotid plaque by CTA-based radiomics: a multicenter study
Source: Front Neurol. 2026 Jan 21;17:1750076. doi: 10.3389/fneur.2026.1750076 (PMC12867918; doi:10.3389/fneur.2026.1750076)
Supplement: Supplementary file 4 [file Table_2.docx]

| **Feature Name** | **Category (Filter_Class)** | **IBSI-Compliant Interpretation** | ****Contextual Interpretation for Carotid Plaque Vulnerability**** |
| --- | --- | --- | --- |
| wavelet-HLH_firstorder_Mean | First-order | The average voxel intensity within the volume of interest, calculated on an image filtered with a specific wavelet (HLH) bandpass filter. | Reflects the **average density of a predominant tissue component** at a specific spatial frequency scale, which provides a quantitative measure of overall plaque composition at this scale. |
| log-sigma-3-0-mm-3D_glszm_SizeZoneNonUniformityNormalized | GLSZM (Texture) | A normalized measure of the variability in the sizes of connected regions (zones) of homogeneous intensity. Low values indicate a uniform distribution of zone sizes. | High values suggest a **heterogeneous mix of small and large tissue clusters**, indicating structural disorganization, associated with plaque instability. |
| squareroot_glcm_MCC | GLCM (Texture) | Maximal Correlation Coefficient. Derived from the square root of the second largest eigenvalue of the GLCM, it measures the complexity and magnitude of gray-level dependencies in an image. | High values may indicate **intricate, complex tissue patterns**, possibly reflecting a chaotic arrangement of components like fibrous tissue, lipid, and calcification. |
| squareroot_glcm_ClusterShade | GLCM (Texture) | A measure of the skewness and uniformity of the GLCM. It assesses the asymmetry of the matrix. Values near zero indicate symmetry, while high positive or negative values indicate asymmetry. | Quantifies **textural asymmetry**. A significant deviation from zero may indicate a **dominant presence of either very high-attenuation or very low-attenuation regions**, suggesting compositional bias. |
| lbp-3D-m2_firstorder_Minimum | First-order | The minimum voxel intensity value within the volume of interest, calculated on an image transformed with a specific 3D Local Binary Pattern (LBP) filter. | Identifies the **darkest voxels within local micro-texture patterns**, potentially pinpointing **small, focal areas of lipid or hemorrhage** that are key vulnerability markers. |
| log-sigma-3-0-mm-3D_glcm_ClusterShade | GLCM (Texture) | A measure of GLCM skewness and asymmetry, calculated on an image filtered with a Laplacian of Gaussian filter (σ=3mm) to highlight blob-like structures of a specific size. | Measures **asymmetry in the distribution of larger tissue components** (~3mm scale), such as the relative placement of a large lipid core or calcified nodule. |
| wavelet-HHL_firstorder_Minimum | First-order | The minimum voxel intensity value within the volume of interest, calculated on an image filtered with a specific wavelet (HHL) bandpass filter. | Captures the **most hypodense points at specific texture edges**, possibly indicating **thin fibrous cap regions or juxta-luminal lipid**, critical for rupture risk. |
| wavelet-HHH_glszm_ZonePercentage | GLSZM (Texture) | The fraction of the number of connected regions (zones) relative to the total number of voxels in the volume of interest. | Indicates **textural coarseness at a fine scale**. Low values suggest few large zones, high values suggest many small zones. |
| lbp-3D-k_glszm_GrayLevelVariance | GLSZM (Texture) | The variance in gray-level intensity for the connected regions (zones) of homogeneous intensity within the volume of interest, calculated on an LBP-filtered image. | Measures **local heterogeneity within micro-texture patterns**. High values indicate a "mottled" composition where small, contiguous tissue patches have very different densities. |
| exponential_glszm_GrayLevelVariance | GLSZM (Texture) | The variance in gray-level intensity for the homogeneous zones, calculated on an exponentially transformed image. This transformation enhances contrast in lower intensity ranges. | Quantifies **heterogeneity specifically within the soft, low-attenuation plaque components**. |
| gradient_firstorder_Maximum | First-order | The maximum value of the gradient magnitude within the volume of interest. The gradient magnitude matrix highlights local rates of intensity change (edges). | Captures the **sharpest intensity transition** in the plaque, which could correspond to the **fibrous cap-lumen interface or the edge of a large calcification**. |
| exponential_glcm_ClusterShade | GLCM (Texture) | A measure of GLCM skewness and asymmetry, calculated on an exponentially transformed image. This provides heightened sensitivity to variations in lower intensities. | Assesses textural asymmetry specifically within low-attenuation plaque components. This may reflect an asymmetric spatial distribution of vulnerable elements, such as lipid or intraplaque hemorrhage, within the plaque architecture. |

****Abbreviations:**** GLCM: Gray-Level Co-occurrence Matrix; GLSZM: Gray-Level Size Zone Matrix; LBP: Local Binary Pattern.

**IBSI Reference:** Zwanenburg A, Vallières M, Abdalah MA, et al. The Image Biomarker Standardization Initiative: Standardized Quantitative Radiomics for High-Throughput Image-based Phenotyping. Radiology. (2020);295(2):328-338. doi:10.1148/radiol.2020191145
