# Supplementary figures and images for: Identification of symptomatic carotid plaque by CTA-based radiomics: a multicenter study
Source: Front Neurol. 2026 Jan 21;17:1750076. doi: 10.3389/fneur.2026.1750076 (PMC12867918; doi:10.3389/fneur.2026.1750076)

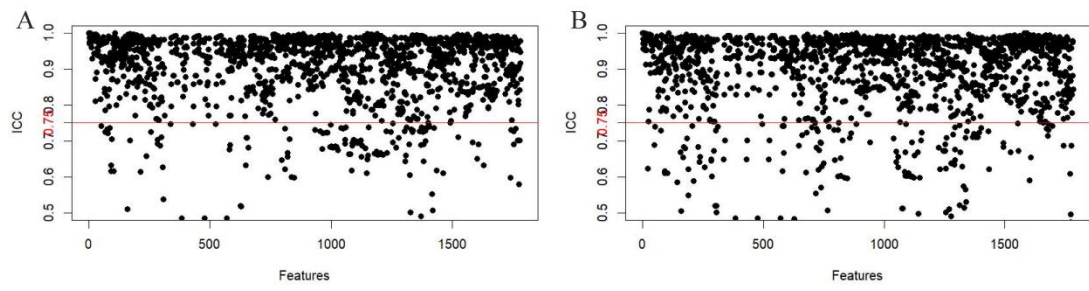

The results of (A) intra-observer consistency and (B) inter-observer consistency assessment.

Supplement: Supplementary file 5 [file Image_1.pdf]
